# Multiparametric autoantibody analysis: a new paradigm for the diagnosis of connective tissue diseases

**DOI:** 10.1186/s13075-022-02980-x

**Published:** 2022-12-23

**Authors:** Nicola Bizzaro, Danilo Villalta, Vittorio Bini, Paola Migliorini, Franco Franceschini, Silvia Piantoni, Emirena Garrafa, Valeria Riccieri, Antonella Fioravanti, Francesca Bellisai, Marilina Tampoia, Marco Fornaro, Florenzo Iannone, Anna Ghirardello, Margherita Zen, Boaz Palterer, Paola Parronchi, Maria Infantino, Maurizio Benucci, Amelia Rigon, Luisa Arcarese, Stefania Del Rosso, Valentina Canti, Elena Bartoloni, Roberto Gerli, FIRMA Collaborators, Onelia Bistoni, Giacomo Cafaro, Carlo Perricone, Fabiana Topini, Ilaria Cavazzana, Micaela Fredi, Stefania Masneri, Sara Cheleschi, Maria-Romana Bacarelli, Daniele Cammelli, Mariangela Manfredi, Roberto Giacomelli, Patrizia Rovere Querini

**Affiliations:** 1 Laboratory of Clinical Pathology, San Antonio Hospital, Tolmezzo, Italy; 2 Azienda Sanitaria Universitaria Integrata di Udine, Udine, Italy; 3 Immunology and Allergology, S. Maria degli Angeli Hospital, Pordenone, Italy; 4 Internal Medicine, Endocrine and Metabolic Science Section, University of Perugia, Perugia, Italy; 5 Clinical Immunology, University of Pisa, Pisa, Italy; 6 Rheumatology and Clinical Immunology Unit, Department of Clinical and Experimental Sciences, ASST Spedali Civili and University of Brescia, Brescia, Italy; 7 Laboratory of Clinical Chemistry, Department of Molecular and Translational Medicine, ASST Spedali Civili and University of Brescia, Brescia, Italy; 8 Rheumatology, “Sapienza” University, Rome, Italy; 9 Rheumatology Unit, Department of Medicine, Surgery and Neuroscience, Azienda Ospedaliera Universitaria Senese, Policlinico Le Scotte, Siena, Italy; 10 Clinical Pathology, Presidio Ospedaliero SS. Annunziata, Taranto, Italy; 11 Rheumatology Unit, Department of Emergence and Transplantation (DETO), University of Bari, Bari, Italy; 12 Rheumatology Unit, Department of Medicine, University of Padua, Padua, Italy; 13 Department of Experimental and Clinical Medicine, University of Florence, Florence, Italy; 14 Laboratory of Immunology and Allergology, San Giovanni di Dio Hospital, Florence, Italy; 15 Rheumatology Unit, San Giovanni di Dio Hospital, Florence, Italy; 16 Clinical Immunology and Rheumatology, University Campus Biomedico, Rome, Italy; 17 IRCCS San Raffaele, Milan, Italy; 18 Rheumatology Unit, Department of Medicine and Surgery, University of Perugia, Perugia, Italy

**Keywords:** Connective tissue diseases, Multiplex testing, Autoantibody profiling, Particle-based multi-analyte technology

## Abstract

**Background:**

In patients affected by connective tissue diseases (CTDs), the identification of wide autoantibody profiles may prove useful in early diagnosis, in the evaluation of prognosis (risk stratification), and in predicting response to therapy. The aim of the present study was to evaluate the utility of multiparametric autoantibody analysis performed by a new fully automated particle-based multi-analyte technology (PMAT) digital system in a large multicenter cohort of CTD patients and controls.

**Methods:**

Serum samples from 787 patients with CTD (166 systemic lupus erythematosus; 133 systemic sclerosis; 279 Sjögren’s syndrome; 106 idiopathic inflammatory myopathies; 103 undifferentiated CTD), 339 patients with other disorders (disease controls) (118 infectious diseases, 110 organ-specific autoimmune diseases, 111 other rheumatic diseases), and 121 healthy subjects were collected in 13 rheumatologic centers of the FIRMA group. Sera were analyzed with the Aptiva-PMAT instrument (Inova Diagnostics) for a panel of 29 autoantibodies.

**Results:**

Multiparametric logistic regression showed that enlarged antibody profiles have a higher diagnostic efficiency than that of individual antibodies or of antibodies that constitute classification criteria for a given disease and that probability of disease increases with multiple positive autoantibodies.

**Conclusions:**

This is the first study that analyzes the clinical and diagnostic impact of autoantibody profiling in CTD. The results obtained with the new Aptiva-PMAT method may open interesting perspectives in the diagnosis and sub-classification of patients with autoimmune rheumatic diseases.

## Introduction

Connective tissue diseases (CTDs) represent a group of heterogeneous disorders, involving multiple body systems. Antinuclear antibodies (ANA) can be associated with various CTDs, including systemic lupus erythematosus (SLE), primary Sjögren’s syndrome (pSS), systemic sclerosis (SSc), idiopathic inflammatory myopathies (IIM), mixed connective tissue disease (MCTD), and undifferentiated connective tissue disease (UCTD). As these diseases, especially in the initial phase, may have many overlapping clinical features and be therefore not easily distinguishable based on symptoms alone, ANA specificities are important biomarkers for the differential diagnosis. For this reason, some specific ANA are included in the classification criteria of CTD, such as anti-Ro60 for pSS [1], anti-topoisomerase I (also known as Scl70), anti-centromere B protein (CENP-B), and anti-RNA polymerase III (RNA pol III) for SSc [2]; anti-histidyl tRNA synthetase (Jo1) for IIM [3]; anti-U_1_RNP for MCTD [4]; and anti-dsDNA as well as anti-Sm for SLE [5]. In addition, ANA represent the entry criterion in the 2019 European League Against Rheumatism/American College of Rheumatology (EULAR/ACR) classification criteria for SLE [5]. However, it is important to remark that ANA are useful not only to classify CTD, but also to diagnose the diseases at a very early stage, where the patient could benefit from early therapeutic intervention.

While the pathogenic role of ANA remains largely unclear, in some CTDs, as in SSc and IIM, specific ANA not only represent important diagnostic tools, but also help to stratify patients into subsets with different clinical features, treatment response, and disease outcome [6–9]. Finally, anti-dsDNA levels are correlated with SLE activity, in particular with renal involvement [10], making detection and quantitative measurement of such antibodies relevant in monitoring disease course [11].

Traditionally, the indirect immunofluorescence assay using human epithelial type-2 cells (HEp-2 IFA) exhibits high sensitivity and represents a commonly used screening assay for ANA. In the case of a positive ANA result, second-line tests are performed to identify the target antigen(s) [12] using solid phase assays, such as enzyme-linked immunosorbent assays, fluorometric enzyme-linked immunoassays, or chemiluminescence immunoassays [13, 14]. However, the need for testing a growing number of antibody specificities requires the use of multiplexing immunoassays. For this purpose, platforms allowing the simultaneous detection of multiple autoantibodies (i.e., dot and line immunoassays or addressable laser bead immunoassay) have been developed and are largely used in the clinical laboratories [15, 16]. However, all these methods enable the detection of a limited number of autoantibodies, usually between eight and twelve.

Recently, a full automated digital system using particle-based multi-analyte technology (PMAT) has been developed. In this multiplexed assay, each different autoantigen is linked to a unique particle. After incubation of the patient sample, antibody binding is revealed by a camera-based system, thus allowing the simultaneous detection of multiple autoantibody specificity. Recent studies demonstrated the good accuracy of PMAT in the detection of multiple antibodies in autoimmune diseases, such as primary biliary cholangitis [17], IIM [18–20], and anti-phospholipid syndrome (APS) [21–23]. In particular, the PMAT panel designed for anti-phospholipid autoantibodies allowed the identification of patients suspected for APS not fulfilling disease classification criteria by the contemporary detection of multiple anti-phospholipid antibodies, including anti-beta 2 glycoprotein I domain I and anti-phosphatidylserine/prothrombin antibodies [21, 23]. In patients with IIM, PMAT showed a good agreement with the immunoprecipitation assay, which is the reference method to detect myositis-specific antibodies (MSA), and a higher accuracy than line immunoassay [18].

These preliminary results suggest that this novel method may be employed for the simultaneous detection of multiple autoantibodies, improving both diagnostic power and risk stratification in patients according to antibody positivity. However, to date, no study analyzed the performance of PMAT in detecting multiple antibodies in CTD.

Therefore, the aim of the present study was to assess the diagnostic performance of the PMAT system in the detection of a high number of CTD-related autoantibodies in a large multicenter cohort of patients with CTD, compared to controls with infectious diseases, patients with organ-specific autoimmune diseases, patients with other rheumatic diseases, and healthy subjects.

## Materials and methods

### Patients

Serum samples from 1247 subjects collected in 13 different Italian Rheumatology centers of the FIRMA (Forum Interdisciplinare di Ricerca nelle Malattie Autoimmuni) group were analyzed in this study. The series comprised 787 patients with CTD classified according to internationally accepted criteria (701 females/86 males; mean age, 41.3 years; range, 16–88 years): 166 SLE, 133 SSc (52 diffuse cutaneous and 81 limited cutaneous), 279 pSS, 106 IIM, and 103 ANA-positive UCTD. The control group (460 subjects; 293 females/167 males; mean age, 45.9 years; range, 5–88 years) included 339 disease controls: 118 samples from subjects with infectious diseases (27 hepatitis C virus, 21 Epstein-Barr virus, 22 cytomegalovirus, 25 hepatitis B virus, and 23 syphilis), 110 from patients with organ-specific autoimmune diseases (25 autoimmune thyroid diseases, 25 celiac disease, 30 primary biliary cholangitis, and 30 autoimmune gastritis), 111 from patients with other rheumatic diseases (15 rheumatoid arthritis, 23 polymyalgia rheumatica, 17 fibromyalgia, 36 ankylosing spondylitis, 17 psoriatic arthritis, 3 osteoarthritis), and 121 samples from healthy subjects (blood donors). The number of samples to be collected was defined on the basis of the known prevalence of the different antibodies in the different CTDs [24], in order to have a number of positive samples for each one of the most significant antibodies, adequate to obtain statistical significance. In the CTD cohort, 15% were first diagnoses and 85% were patients in follow-up; the mean interval from diagnosis was 7.7 years (range, 0–43).

ANA HEp-2 IFA (performed in each individual site) were positive at a 1:80 titer in 164/166 (98.8%) SLE, in 131/133 (98.5%) SSc, in 249/279 (89.2%) pSS, in 84/106 (79.2%) IIM, and in 207/460 (45.0%) controls. UCTD patients were all ANA positive as per selection criteria.

Samples were coded by the recruiting centers and serological analyses were performed blinded to clinical data.

For each patient, demographic and clinical data were recorded according to the specific pathology. All data was entered in a database and analyzed.

### Methods

Sera were tested on the Aptiva™ instrument (Inova Diagnostics, San Diego, CA; Research Use Only). Aptiva is a digital automated system that uses particle-based multi-analyte technology (PMAT) to simultaneously measure multiple autoantibodies in one single step. The technology is based on the use of a mixture of suspended microparticles that have a unique color code, individually coated with a different antigen. Each unique color code allows the antigens to be identified within the process. After incubation with diluted patients’ sera, particles are washed and incubated with anti-human IgG conjugated to phycoerythrin. Finally, after another washing cycle, particles are aligned in a monolayer and analyzed through digital imaging technology using two LEDs. A first red LED is used to identify the analyte, while a second green LED allows the measurement of the fluorescence intensity. The reaction data are captured digitally by a sophisticated high-resolution charged coupled device (CCD) sensor. The acquired image is subsequently stored in the analyzer database for calculation and release of quantitative results. To verify the correct instrument functionality, the system uses quality control samples that contain antibodies specific for each analyte tested.

### Autoantibody profiles

The samples were centralized in the Pordenone Laboratory of the FIRMA group and tested with the following three multiparametric antigenic panels: CTD IgG Essential™ (dsDNA, DFS70, U_1_RNP, Sm, Ro60, Ro52, La, Scl70, Jo1, CENP-B, Ribo-P), CTD IgG Comprehensive™ (RNA pol III, Th/To, Ku, BICD2, PM/Scl), and Autoimmune Myopathy IgG™ (Mi-2, HMGCR, NXP2, MDA5, PL-7, PL-12, EJ, SRP, TIF1γ, SAE e OJ).

The Aptiva instrument uses ready-to-use cartridges containing all the specific reagents for the analytical reaction, including the mixture of the microspheres coated with the various antigens defined in each profile.

### Statistical analysis

The optimal cutoff for each one of the three antibody panels (CTD Essential, CTD Comprehensive, and Autoimmune Myopathies) was selected by a receiver operating characteristic (ROC) curve analysis and set at 5 arbitrary units (AU)/ml for antibodies included in the CTD Essential profile and 1 AU for both the Comprehensive and Autoimmune Myositis panels.

The diagnostic sensitivity of each antibody was calculated separately in the different CTD; the diagnostic specificity was calculated in the control group (disease controls and healthy subjects). In addition, considering that Aptiva also provides a screening function, indicating whether all the searched antibodies are negative or if at least one of these is positive, we calculated the cumulative diagnostic sensitivity and specificity of all antibodies included in the three profiles across the CTD population.

The distribution of variables was first checked by the Shapiro–Wilk test, and as all the data were non-normally distributed, the Mann–Whitney *U*-test was used for comparisons of variables.

Bivariate and multivariate logistic regression models were fitted for the prediction of patient status incorporating to the final models the variables significant in bivariate analysis and the main clinically predictive ones. Collinearity problems were corrected excluding predictors that caused the model instability. To decrease the overfit bias and internally validate our results, all regressions were subjected to 200 bootstrap resamples and the goodness-of-fit of logistic models was checked using Hosmer and Lemeshow test. The predictive accuracy of logistic regression models was quantified as the area (AUC) under the ROC curve built on the patient probability of being “case” or “control” derived from the logistic regression equation. To determine if an observation should be classified as positive or negative, we chose a cutoff point such that observations with a fitted probability above the cutoff point are classified as positive and any observations with a fitted probability below the cutoff point are classified as negative. The **AUC** gives us an idea of how well the model is able to distinguish between positive and negative outcomes. Predictive accuracies as AUC were compared using the DeLong method.

In order to calculate the probability of disease occurring, the logistic regression formula can be written in a general linear equation form as follows: ln(*P*/(1-*P*)) = *β*_0_+ *β*_1_*X*_1_+ *β*_2_*X*_2_+... where ln is the natural logarithm; *P* the probability of the event; (*P*/(1-*P*) is the odds; *β*_0_, *β*_1_, *β*_2_... are the regression coefficients; and *X1*,*X2*, … are the independent variable values. Solving for the probability equation results in *P* = 1 /(1+*e*^−(*β*0+ *β*1*X*1+ *β*2*X*2+...)^) where *e* is the Euler’s constant 2.718282.

All statistical analyses were performed using IBM-SPSS® version 26.0 (IBM Corp., Armonk, NY, USA, 2019). In all analyses, a two-sided *p*-value <0.05 was considered significant.

## Results

### Diagnostic accuracy of autoantibody profiles

Autoantibody prevalence (diagnostic sensitivity of the PMAT assay) in the different disease groups and the diagnostic specificity of each autoantibody for the panels CTD Essential, CTD Comprehensive, and Autoimmune Myopathies are shown in Table 1.

**Table 1:**
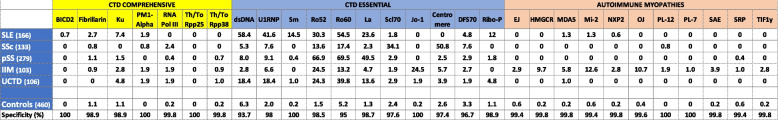
Prevalence (%) of 29 autoantibodies in the different connective tissue diseases and in controls (antibody specificity is also indicated)

*SLE* systemic lupus erythematosus, *SSc* systemic sclerosis, *pSS* primary Sjögren’s syndrome, *IIM* idiopathic inflammatory myositis, *UCTD* undifferentiated connective tissue disease

In the 166 SLE patients, anti-dsDNA autoantibodies were the most frequent finding (58.4%), followed by Ro60 (54.5%), U_1_RNP (41.6%), and Ro52 (30.3%). Anti-Sm antibodies were present in 14.5% of SLE patients. Sixteen (9.4%) patients had no detectable antibodies.

Anti-Ro60 (69.5%), anti-Ro52 (66.9%), and anti-La (49.5%) antibodies were prevalent in Sjögren’s syndrome [no antibodies were detected in 58/279 (20.8%) patients], while in 133 SSc patients, anti-centromere (CENP-B) accounted for 50.8% and anti-Scl70 for 34.1%. When SSc was splitted in the two clinical subsets, namely limited cutaneous (lcSSc) and diffuse cutaneous (dcSSc), of the 81 patients with clinical features of lcSSc, 75.3% were anti-CENP-B positive, 12.3% were anti-Scl70 positive, and 1.2% were anti-RNA pol III positive. Among the 52 patients with clinical features of dcSSc, anti-Scl70 antibodies were detected in 68.6%, anti-RNA pol III in 3.9%, and anti-CENP-B in 9.8%. In both SSc subsets, there was no overlap between anti-CENP-B, Scl70, and anti-RNA pol III antibodies. Thirteen SSc patients (9.8%) had no antibodies.

Myositis-specific antibodies (MSA) were detected in 69/106 (65.1%) of the patients with IIM; of these, 26/69 (37.7%) were anti-Jo1 positive (in 11 patients as the only antibody). Though the prevalence of the other MSA ranged from 1 to 12.6% being these autoantibodies almost mutually exclusive, their specificity always exceeded 99%. Besides MSA, anti-Ro52 which despite not being an MSA is known to associate with IIM [25] were detected in 26/106 (24.5%) patients; in 13 cases, they associated with anti-Jo1; in three cases, with another MSA; and in 10 cases, they were the only antibodies.

In the UCTD group, anti-Ro60 were the most frequent finding (39.8%) followed by anti-Ro52 (24.3%), anti-dsDNA, and anti-U_1_RNP (both 18.4%). A total of 38/103 (36.9%) UCTD patients scored negative for all antibodies.

### Autoantibody profiles in predicting diagnosis

Based on these findings, the best combination of autoantibodies for each disease to predict diagnosis was chosen according to the strength of their diagnostic accuracy in ROC analysis. Multivariate logistic regression models fitted on antibody levels demonstrated the most powerful combination of autoantibodies for each one of the pathology groups.

**Fig. 1:**
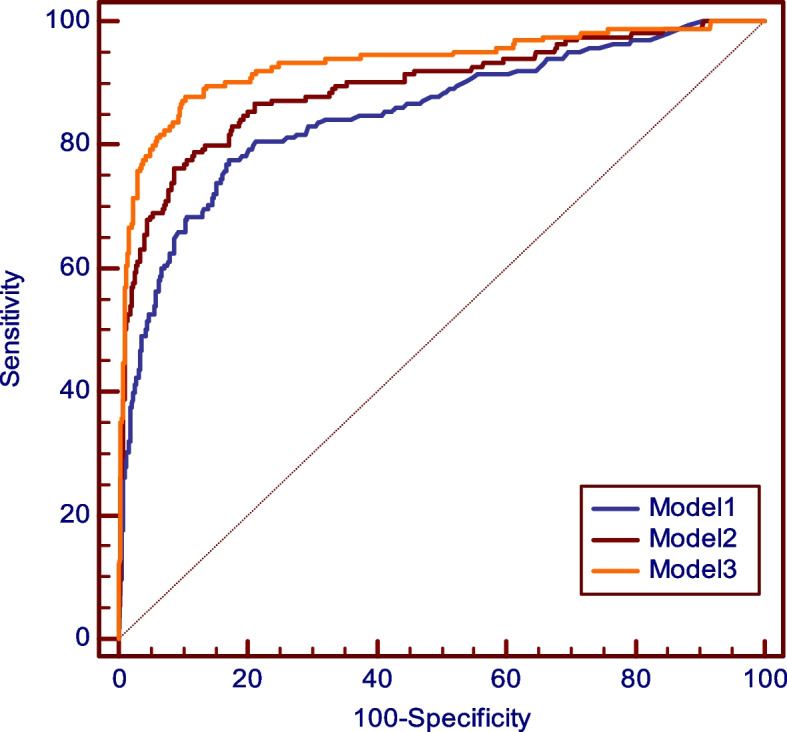
ROC curves for antibodies to dsDNA (model 1), dsDNA + U_1_RNP (model 2), and dsDNA + U_1_RNP + Ro60 (model 3) in SLE. The composite ROC curve of the three antibodies provides an AUC of 0.943

Antibodies to dsDNA (*p*=0.0001), U_1_RNP (*p*=0.001), Ro60 (*p*=0.0001), and ribosomal P (*p*=0.02) were significantly associated with SLE. AUC were 0.849 for dsDNA, 0.880 for U_1_RNP, 0.837 for Ro60, and 0.729 for ribosomal P. When the ROC curves were combined together by adding one more antibody at the time, U_1_RNP + dsDNA provided an AUC of 0.905, and when either Ro60 or ribosomal P was added, the AUC rose to 0.943 (Fig. 1). The addition of both anti-Ro60 and anti-ribosomal P did not gain diagnostic accuracy (AUC 0.942) as all patients with anti-ribosomal P antibodies were also positive either for anti-dsDNA or for U_1_RNP or Ro60 antibodies. Nonetheless, although anti-ribosomal P does not increase diagnostic sensitivity, inclusion of this antibody in the diagnostic profile is important because of its very high specificity for SLE and its association with neuropsychiatric lupus [26].

Anti-Sm, despite the very high specificity (99.8%) and its association with SLE by bivariate logistic regression (*p*<0.001), failed to contribute to a significant increase in the diagnosis of SLE by multivariate logistic regression analysis (*p*=0.19). This may be easily explained by its low sensitivity (14.5%) and because this antibody is found as the only antibody in only a few cases. Indeed, in this series of SLE patients, anti-dsDNA were detected in 78% of the cases, anti-dsDNA + anti-Sm in 20%, and anti-Sm without anti-dsDNA only in 2%.

The same analysis applied to the other diseases showed that in Sjögren’s syndrome there are two antibodies that are significantly associated with: anti-Ro60 and anti-Ro52 (*p*=0.0001 and 0.001, respectively). However, adding anti-Ro52 (AUC 0.850) to anti-Ro60 (AUC 0.837) only slightly improved diagnostic accuracy (AUC 0.869). Though anti-La antibodies showed to be associated to pSS by bivariate logistic regression (*p*<0.001), their association with the disease was not confirmed by multiple regression (*p*=0.238). However, it is important to maintain anti-La antibodies in the diagnostic profile because when anti-Ro60 and anti-La are combined, specificity for Sjögren’s syndrome is high (99.3% vs. controls and 93.4% vs. SLE).

**Fig. 2:**
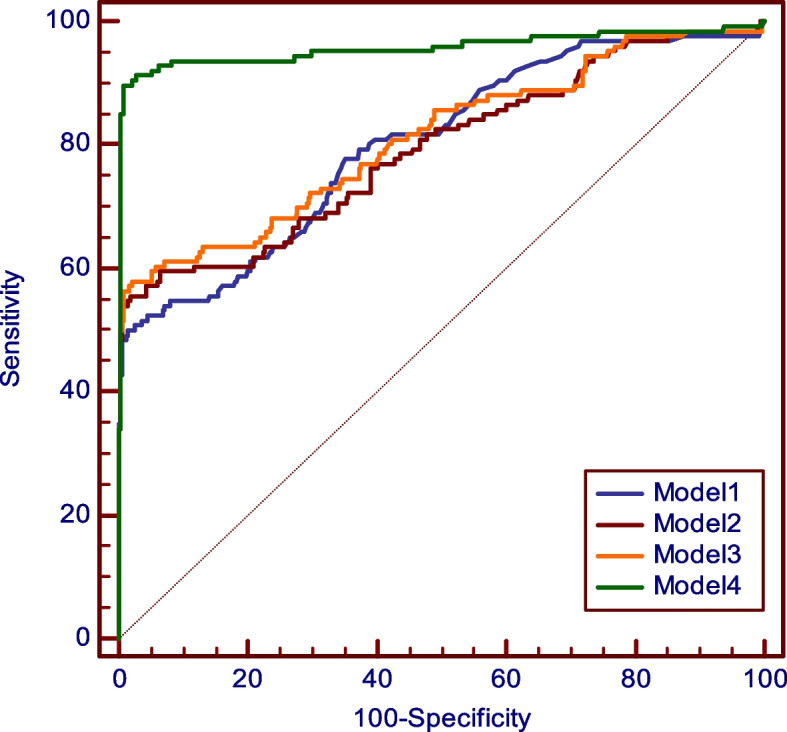
ROC curves for four different models of autoantibody combinations in SSc: CENP-B alone (model 1); CENP-B + RNA pol III (model 2); CENP-B + RNA pol III + U_1_RNP (model 3); CENP-B + RNA pol III + U_1_RNP + Scl70 (model 4). The ROC curve of the four combined antibodies gives an AUC of 0.958

In systemic sclerosis, anti-CENP-B antibodies showed a strong association (*p*=0.0001) with lcSSc (81 patients) and an AUC of 0.902; in dcSSc (52 patients), AUC for anti-Scl70 was 0.874 (*p*=0.001). Anti-RNA pol III and anti-U_1_RNP antibodies were also significantly associated with SSc (*p*=0.012 and *p*=0.001, respectively). Composite ROC curves made up by the combination of four antibodies (anti-CENP-B, anti-Scl70, anti-RNA pol III, and anti-U_1_RNP) yielded the highest AUC value (0.958) (Fig. 2).

**Fig. 3:**
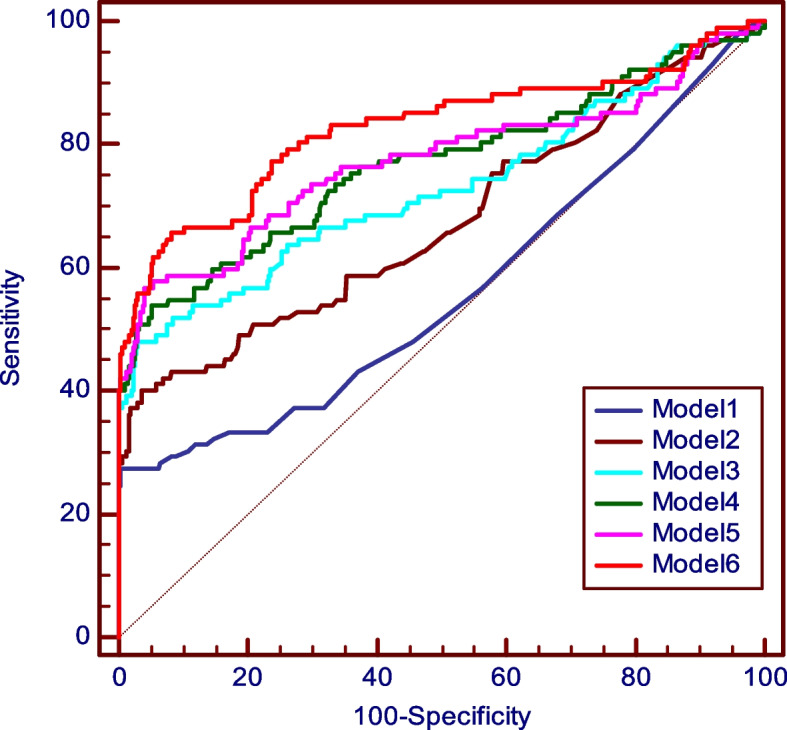
ROC curves for six different models of autoantibody combinations in autoimmune myositis: Jo1 alone (model 1); Jo1 + HMGCR (model 2); Jo1+ HMGCR + MDA5 (model 3); Jo1+ HMGCR + MDA5 + NXP2 (model 4); Jo1 + HMGCR + MDA5 + NXP2 + TIF1γ (model 5); Jo1 + HMGCR + MDA5 + NXP2 + TIF1γ + Ro52 (model 6). The ROC curve of the six combined antibodies provides a global AUC of 0.813

In the group of idiopathic inflammatory myopathies, anti-HMGCR (*p*=0.019), anti-MDA5 (*p*=0.020), anti-NXP2 (*p*=0.020), and anti-Tif1γ (*p*=0.041) were significantly associated with the disease. All MSA combined provided an AUC of 0.701. When anti-Jo1 (composite AUC 0.762) and anti-Ro52 were added, a cumulative AUC of 0.813 was obtained (Fig. 3).

When antibodies included in the three CTD profiles were considered collectively and Aptiva results were used as a screening test, the diagnostic sensitivity of the complete antibody profile in identifying a CTD (UCTD excluded) was 85.7%, much higher therefore than the sensitivity of any antibody taken individually, and the specificity was 87.5% (cumulative AUC was 0.873). Compared to the classical ANA test, at a titer 1:80 HEp-2 IFA, Aptiva was less sensitive (85.7% vs. 91.8%) (*p*=0.0004) but much more specific (77.0% vs. 55%) (*p*<0.0001). At a titer HEp-2 IFA 1:160, Aptiva was slightly more sensitive (85.7% vs. 82.5%), even if not in a statistically significant way (*p*=0.215), and still more specific (77.0% vs. 65.6%) (*p*=0.0003).

### Autoantibody profiles in assessing the probability of disease

We further evaluated the probability of disease diagnosis according to a combination of antibodies (measured as positive/negative), by adding one antibody at a time, chosen among those that were significantly associated to each disease by logistic regression. As shown in Table 2, this model provides a theoretical probability of disease when more autoantibodies are associated in a single subject. For instance, the probability for SLE is 42.3% when only anti-dsDNA antibodies are present, which rises to 52.1% when anti-Sm are also positive and to 96.9% if anti-U_1_RNP are also detected. In pSS, the probability is 42.8% if anti-Ro60 are positive, and reaches a value of 95.6% when Ro52 are also present. In IIM, the probability is 89.1% with positive anti-Jo1 antibodies, reaching 99.7% if another MSA is associated and 100% if anti-Ro52 is also present. In SSc, the analysis was performed in the two clinical forms of the disease, namely, lcSSc and dcSSc, taking into account that anti-CENP-B, anti-Scl70, and anti-RNApIII antibodies are usually mutually exclusive. In lcSSc, the presence of anti-CENP-B antibodies gives a probability of 85.3%; if four antibodies (anti-CENP-B, anti-fibrillarin, anti-PM1alfa, and anti-U_1_RNP) are contemporary present, the probability rises to 99.8%. Similarly, in dcSSc, anti-Scl70 alone provide a probability of 79.2%, increasing to 99.2% when four SSc-related antibodies are detected. The RNApIII-positive group shows a probability of 82.3% if only this antibody is present, increasing to 86.1%, 96.2%, and 98.6% if one, two, or three more antibodies are present simultaneously.

**Table 2:** Disease probability according to multiple antibody presence in CTD

	Autoantibody combination	Probability (%)		Autoantibody combination	Probability (%)
**SLE**	dsDNA alone	42.3	**lcSSc**	CENP-B alone	85.3
	dsDNA+Sm	52.1		CENP-B+Fibrillarin	93.3
	dsDNA+Sm+ U_1_RNP	96.9		CENP-B+Fibrillarin+PM1 alfa	99.1
	dsDNA+Sm+ U_1_RNP+Ro60	99.6		CENP-B+Fibrillarin+PM1 alfa+U_1_RNP	99.8
	dsDNA+Sm+ U_1_RNP+Ro60+Rib P	99.8			
	dsDNA+Sm+ U_1_RNP+Ro60+Rib P+Ro52	100	**dcSSc**	Scl70 alone	79.2
				Scl70+Fibrillarin	87.3
**IIM**	Jo1 alone	89.1		Scl70+Fibrillarin+PM1 alfa	97.4
	Jo1+MSA	99.7		Scl70+Fibrillarin+PM1 alfa+U_1_RNP	99.2
	Jo1+MSA+Ro52	100			
			**lcSSc or dcSSc**	RNApIII alone	82.3
**pSS**	Ro60 alone	42.8		RNApIII +Fibrillarin	86.1
	Ro60+Ro52	95.6		RNApIII +Fibrillarin+PM1 alfa	96.2
	Ro60+Ro52+La	98.9		RNApIII +Fibrillarin+PM1 alfa+U_1_RNP	98.6

*SLE* systemic lupus erythematosus, *SSc* systemic sclerosis, *lcSSc* limited cutaneous SSc, *dcSSc* diffuse cutaneous SSc, *pSS* primary Sjögren’s syndrome, *IIM* idiopathic inflammatory myositis, *UCTD* undifferentiated connective tissue disease

## Discussion

The search for autoantibodies when there is a clinical suspicion of CTD is a tool of great importance for diagnostic purposes because many antibodies are associated with well-defined CTDs and above all because in the initial stages of the disease, signs and symptoms do not allow to point towards a specific disease [27]. Traditional laboratory tests have analyzed one protein at a time giving much if not exclusive importance to autoantibodies that constitute classification-diagnostic criteria for the different CTD. As a paradigmatic feature of CTD is the presence of multiple autoantibodies [28–30], measurement of autoantibody profiles can give an important contribution in the early diagnosis of CTD. A classic example is SLE, the prototype of CTD, in which almost 180 different autoantibodies have been described [31]. Another illustrative example is the case of patients with autoimmune myositis. Rather than being one homogenous group, the multiple autoantibodies seen in autoimmune myositis are now thought to correlate with specific subtypes with different clinical features [32]. Thus, antibody profiles rather than individual tests may provide higher diagnostic sensitivity and specificity. In addition, their detection can be very useful for prognostic purposes and risk stratification [33]. In this case, the clinical implications are evident, allowing more accurate and timely monitoring of each individual patient, representing a concrete step towards the application of personalized medicine. However, the available technologies do not provide for the measurement of an extended number of autoantibodies, and performing multiple antibody profiles is possible only using two or more analytical methods, with increasing costs and time. In this study, we evaluated and validated a new analytical method based on particle multi-analyte technology capable of rapidly measuring a large number of autoantibodies, in a wide range of subjects with well-characterized CTD and in numerous control samples.

The results showed that the PMAT method has a very high specificity, between 93.7 and 100% in the detection of 29 autoantibodies which are markers of CTD. This is a finding of great importance as high specificity is mandatory when multiple antibodies are measured simultaneously because the risk of false positive results increases progressively with the increase in the number of antibodies that are measured [30]. In this cohort, the lowest specificity (93.7%) was observed for anti-dsDNA antibodies. Though this value as calculated against disease and healthy controls fully complied with the requirements of the EULAR/ACR criteria [5], they were also detected in 8% of the 279 patients with Sjögren’s syndrome and in 5.3% of patients with SSc, without overlapping clinical feature with SLE. Accurate measurement of anti-dsDNA antibodies is a well-known issue [34] and, due to their great heterogeneity and polyclonality of the autoimmune response to native DNA in individual patients, largely depends on the detection method [35].

The objective of this study was not only to evaluate and validate the performance of the new analytical method, verifying its diagnostic accuracy, but above all to verify if it was useful to investigate a larger antibody panel than that which is normally sought in clinical laboratories. Therefore, logistic regression models were complemented by predictive accuracy tests. Using logistic regression analysis for quantitative antibody values, we demonstrated that diagnostic AUC for each one of the CTDs considerably improved by adding antibodies in a step-wise fashion. In SLE, AUC raised to the highest value (0.943) when four antibodies were considered (anti-dsDNA, anti-Sm, anti-U_1_RNP, and anti-Ro60 or anti-ribosomal P). The same was observed for pSS (AUC 0.867), SSc (AUC 0.958), and IIM (AUC 0.813). These high AUC values show that the use of antibody profiles improves the diagnostic accuracy of immunological tests.

Based on test results, we have also proposed a mathematical model to calculate the probability of disease diagnosis independently from antibody values, i.e., only considering dichotomic (positive/negative) results. Data showed that combining positive results for different antibodies provides an increasing probability of disease, thus adding a significant value of antibody profiles to the clinical diagnosis.

Another important issue is related to the possible use of the Aptiva system as a screening tool. The use of an antibody profile that includes all the main antibodies associated with CTD can be extremely useful in the early stages of the disease, when clinical findings are blurred and do not yet allow to point towards a specific disease. In this series, considering samples to be positive when at least one antibody is detected, only 14.3% of the CTD samples scored negative for all antibodies, providing a cumulative diagnostic sensitivity of the three profiles of 85.7%. This figure is lower than that obtained by the classical ANA HEp-2 IFA test at 1:80 dilution (92%), but similar to that obtained with HEp-2 IFA at 1:160 dilution (82.5%) and much more specific at both dilutions (77.0% vs. 54.3% at 1:80 and vs. 66.1% at 1:160).

The limit of this study is that the results were obtained evaluating selected and well-characterized cohorts of subjects with CTD and this may be not completely reproducible in a real-life series of diagnostic patients. However, for the purposes of this study, it was essential that the serum samples came from subjects that fully met the classification criteria of the respective diseases, to avoid possible misinterpretation of test results. Another limitation regards the control group. Though it comprehended a very large number of subjects with an ample heterogeneity of diseases, further studies are needed to confirm the very high specificity of the PMAT-Aptiva immunoassay observed in this study, also in subjects with autoimmune rheumatic disorders not included in the present study, such as ANCA-associated vasculitis and the anti-phospholipid syndrome.

## Conclusions

This is the first study that has measured so many autoantibodies in CTD outside the research field by a novel technology now available to clinical laboratories. We found that the CTD Essential™ panel was adequate to diagnose SLE and pSS. The combination of the panel CTD Essential™ plus CTD Comprehensive™ yields the best results for SSc diagnosis, and the Autoimmune Myositis™ panel complemented by Ro52 was the optimal antibody profile to diagnose IIM. However, since in the early phase of CTD, identifying a specific disease is not easy, a profile extended to three panels can be an acceptable and advantageous solution. Moreover, it should be noted that none of the 29 autoantibodies tested in this profile could be detected in 14.3% of the patients in this series. Other very specific antibodies can therefore possibly be added in the future to fill the residual diagnostic gap.

## Data Availability

Data analyzed during the current study are available from the corresponding author on reasonable request.

## Abbreviations

ACR: American College of Rheumatology
ANA: Antinuclear antibodies
AUC: Area under the curve
BICD2: Bicaudal D homolog 2
CENP-B: Centromere B protein
CTD: Connective tissue disease
EULAR: European League Against Rheumatism
HMGCR: 3-Hydroxy-3-methylglutaryl coenzyme A reductase
IFA: Immunofluorescence assay
IIM: Idiopathic inflammatory myopathies
MCTD: Mixed connective tissue disease
MDA5: Melanoma differentiation-associated gene 5
MSA: Myositis-specific antibodies
NXP2: Nuclear matrix protein 2
PMAT: Particle-based multi-analyte technology
pSS: Primary Sjögren’s syndrome
SAE: Small ubiquitin-like modifier activating enzymes
SLE: Systemic lupus erythematosus
SRP: Signal recognition particle
SSc: Systemic sclerosis
dcSSc: Diffuse cutaneous systemic sclerosis
lcSSc: Limited cutaneous systemic sclerosis
TIF1γ: Transcription intermediary factor gamma
UCTD: Undifferentiated connective tissue disease
